# Oral Health: A Major Global Public Health Concern

**DOI:** 10.3390/jcm14124101

**Published:** 2025-06-10

**Authors:** Frédéric Denis, Céline Clement

**Affiliations:** 1 Faculty of Dentistry, University of Tours, 37032 Tours, France; celine.clement@univ-tours.fr; 2 Division of Education, Ethics, Health, Faculty of Medicine, University of Tours, 37044 Tours, France; 3 Department of Medicine and Bucco-Dental Surgery, Tours University Hospital, 37044 Tours, France

Oral diseases rank among the most prevalent noncommunicable diseases (NCDs) worldwide, affecting approximately 3.5 billion people. These conditions share several modifiable risk factors with other NCDs, including excessive sugar intake, tobacco use, and harmful alcohol consumption, in addition to common social and environmental determinants [1]. Given that they are largely preventable, oral diseases require targeted strategies focused on mitigating these risk factors.

Effective prevention involves reducing exposure to these risks, early detection of carious and periodontal lesions, and timely treatment to halt disease progression. Adequate oral hygiene is a cornerstone of prevention: regular brushing and interdental cleaning disrupt dental plaque, a biofilm composed of bacteria and their by-products adhering to teeth and prosthetic devices. In the absence of proper hygiene, this biofilm evolves into microbial dysbiosis, increasing its pathogenic potential.

This microbial imbalance can trigger a hematogenous dissemination of bacteria and inflammatory mediators, thereby amplifying systemic inflammation in susceptible individuals. Converging evidence links these processes to multisystemic effects, particularly involving the endocrine, cardiovascular, pulmonary, and neurological systems [2].

This Special Issue contributes to ongoing global efforts led by the World Health Organization (WHO), including the “Global Strategy and Action Plan on Oral Health 2023–2030” [3] and the first-ever global meeting on oral health held in Bangkok in November 2024 [4]. During this event, delegations from over 110 countries developed national roadmaps and a joint declaration. As emphasized by WHO Director-General Dr. Tedros Adhanom Ghebreyesus, “Oral health is an essential component of well-being… The WHO calls on all countries to prioritize prevention and expand access to affordable oral health care…”. Henceforth, “There is no health without oral health”—a declaration adopted in Bangkok on 29 November 2024.

This Special Issue highlights several critical topics, such as the importance of high-quality interdisciplinary and transdisciplinary academic training on the relationship between human papillomavirus (HPV) infection, oral lesions, and the role of vaccination in preventing HPV-related diseases [5]. Enhancing dentists’ knowledge of the symptoms, clinical stages, and pharmacological treatment—including its adverse effects—of Alzheimer’s disease is equally essential. Supporting caregivers and ensuring adequate funding and resources are necessary to assist institutionalized patients or nursing home residents in maintaining daily oral hygiene [6].

Innovative approaches, such as interdental cleaning splints, may prove beneficial for individuals with functional impairments or anatomical limitations that hinder effective oral hygiene. These tools are especially promising given the growing interest of younger populations in digital health technologies [7,8]. However, high-quality studies are needed to rigorously evaluate the effectiveness of these digital health interventions [8].

In addition, oral health among incarcerated populations remains under-researched, despite its potential contribution to social reintegration—particularly among women [9]. Likewise, the trajectory of dental caries in children with coronary heart disease remains poorly understood due to methodological limitations in existing studies [10].

These findings underscore the persistent knowledge gaps in oral health promotion and prevention. Therefore, a meaningful integration of oral health into public health agendas is imperative, in alignment with WHO recommendations [11].

In France, the National Health Conference (Conférence nationale de santé, CNS) and the Directorate-General for Health have issued a series of recommendations aimed at incorporating oral health into the National Health Strategy (Stratégie nationale de santé, SNS), including the following:

Establishing a strong political leadership through the appointment of a national interministerial delegate for oral health to coordinate an ambitious prevention and care policy;

Reducing care pathway discontinuities by improving access to care for vulnerable populations (e.g., individuals in precarious situations or with disabilities);

Expanding prevention efforts in community settings through proactive outreach and full participation of the populations concerned;

Leveraging social media to disseminate public health information and advice, as promoted in the WHO’s “M-Oral Health” program;

Controlling excess fees, which remain a major barrier to access;

Addressing commercial determinants of health by taxing high-risk products (added sugars, tobacco, alcohol), with revenues allocated to fund oral health prevention programs [12].

This Special Issue aims to broadly engage public health stakeholders in addressing the critical yet often overlooked issue of oral health. It seeks to contribute to the development and advancement of public health policies in this field, both at the national and international levels, in line with the recommendations put forward by the WHO.
